# Allergic Contact Dermatitis to Terminalia Chebula Fruit Extract in Antiaging Creams

**DOI:** 10.1111/cod.70060

**Published:** 2025-11-19

**Authors:** Maël Blanchard, Ophélie Marchal, Gabriela Blanchard, Pierre Piletta‐Zanin

**Affiliations:** ^1^ Dermatology Department Geneva University Hospital Geneva Switzerland

**Keywords:** allergic contact dermatitis, case report, *Terminalia chebula*

Allergic contact dermatitis (ACD) is the most common cause of eyelid dermatitis [1]. Cosmetic creams are a major source of eyelid ACD cases, due to contact allergens such as fragrances, preservatives, emulsifiers and nickel. The cosmetics industry has long relied on natural plant resources to discover new active ingredients. We report the first case of eyelid ACD to Terminalia Chebula Fruit Extract in a cosmetic night cream.

## 1 Case Report

**FIGURE 1:**
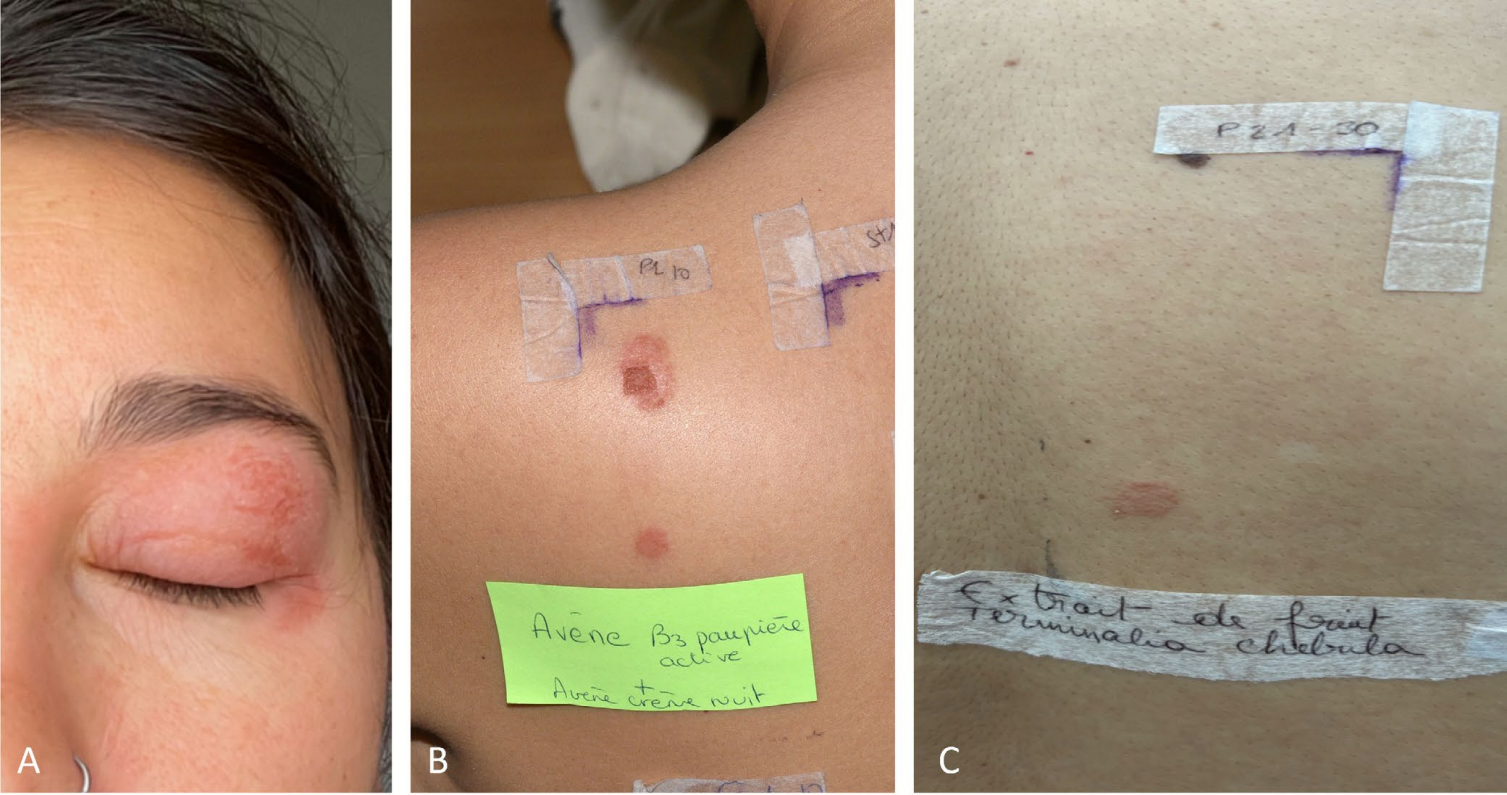
(A) Eyelid allergic contact dermatitis in our patient. (B) Positive patch test on D4 to Hyaluron Activ B3 triple correction eye cream (+++) and Hyaluron Activ B3 multi‐intensive night cream (+). (C) Positive patch test on D4 to *Terminalia chebula* (*++*).

A 31‐year‐old Caucasian atopic female developed a recurrent, well‐demarcated, itchy eyelid (Figure 1A) and lip erythema. Patch tests were performed using the European baseline, preservative and eyelid series (Chemotechnique Diagnostics, Vellinge, Sweden) using IQ Ultra chambers (Chemotechnique Diagnostics) and the patient's own products (‘as is’). Allergens were applied for 48 h. Readings on day (D) 2 and D4 showed positive reactions for Hyaluron Activ B3 triple correction eye cream (+++) and Hyaluron Activ B3 multi‐intensive night cream (+) (Avène, France) (Figure 1B). The European baseline, preservative and eyelid series were all negative except for a +++ positive reaction to paraphenylenediamine (PPD), which was not considered relevant to the current skin reactions. Indeed, the patient presented a strong response to a henna tattoo during adolescence.

Thus, we hypothesized that the personal creams showing a positive reaction, likely contained a common allergen that was not detected in our standard test panels. The individual ingredients of both, Hyaluron Activ B3 triple correction eye cream and multi‐intensive night cream, kindly provided by the manufacturer, were subsequently patch‐tested. They showed a positive reaction (D2+, D4++) to a single ingredient, Terminalia Chebula Fruit Extract (Figure 1C). Five unexposed controls were negative to this extract.

## 2 Discussion

Positive patch tests for both Hyaluron Activ B3 triple correction eye cream and multi‐intensive night cream and for only one of their ingredients confirm the diagnosis of ACD caused by Terminalia Chebula Fruit Extract. Amine‐based PPD and phenolic compounds from this fruit share an aromatic ring structure as part of their molecular backbone, but we did not identify any direct structural similarity between them. *T. chebula* Retz. is an Asian plant from the Combretaceae family which is commonly used in Tibetan and Ayurvedic medicine for various indications [2, 3], especially antioxidant activity. The topical use has been suggested to treat tinea corporis [4], as well as for anti‐aging purposes [5] with an overall good toxicity profile [2]. To date, more than one hundred compounds have been identified for its fruit's extracts, including phenolic acids, tannins, lignans, triterpenoids, flavonoids and volatiles. Among others, gallate esters (derivatives from gallic acid), ellagic acid and caffeic acid could potentially be implicated given their already documented allergenic potential [6, 7, 8]. To the best of our knowledge, ACD in response to Terminalia Chebula Fruit Extract, or any extract derived from other parts of the *T. chebula* plant, has not been previously documented. Its use may increase in a wide range of cosmetic products, and we expect additional cases of ACD due to Terminalia Chebula Fruit Extract to be encountered in the future. Our case emphasises the need to identify new emerging allergens, especially “natural” ones, through testing individual ingredients from personal products.

## Author Contributions

Conceptualization: Maël Blanchard and Pierre Piletta‐Zanin. Data handling: Maël Blanchard and Ophélie Marchal. Manuscript development: Maël Blanchard. Critical revision: Ophélie Marchal, Gabriela Blanchard, and Pierre Piletta‐Zanin.

## Consent

The authors obtained written consent from patients for their photographs and medical information to be published in print and online, with the understanding that this information may be publicly available. Patient consent forms were not provided to the journal but are retained by the authors.

## Conflicts of Interest

The authors declare no conflicts of interest.

## Data Availability

The data that support the findings of this study are available from the corresponding author upon reasonable request.
